# Physical Education and Sports: A Backbone of the Entire Community in the Twenty-First Century

**DOI:** 10.3390/ijerph19127296

**Published:** 2022-06-14

**Authors:** Jean de Dieu Habyarimana, Etienne Tugirumukiza, Ke Zhou

**Affiliations:** 1 Department of Physical Education and Sports Training, School of Physical Education, Minglun Campus, Henan University, Kaifeng 475001, China; jdhdieu@yahoo.fr; 2 Department of Physical Education and Sports Coaching, School of Physical Education and Sports Coaching, Shanghai University of Sport, Shanghai 200433, China; etiennetugira12@gmail.com

**Keywords:** Physical Education and Sports, cognitive, physical, affective, health, social, moral, culture, SDGs

## Abstract

The current state of physical inactivity of people can be traced back to the people who have been denied their fundamental human right to physical education and participation in school sports (PES). Growing up without the fundamental human right to free movement and participation in sports activities enabled students to stay physically inactive. The purpose of this study was to explore what is currently known about the role of PES in all areas of human development and SDGs and to raise awareness about PES, which has been shown to be on the decline. To increase the study’s overall efficacy, an external desk research approach was employed to gather relevant information published online: reports, policies, charters, recommendations, and other relevant articles from various electronic databases and websites of international organizations responsible for PES, culture, and health. PES benefits are discussed in all domains of human development, including physical and mental health, cognitive, psychosocial, and moral benefits. Contrary to its importance to human growth as a whole, PES has been sidelined since the end of the twentieth century. An awareness of the subject of PES has thus been raised as a backbone of the entire community in the twenty-first century, so as to translate the promises and policies of PES into realities and practices.

## 1. Introduction

One of the most significant current discussions in physical exercises and public health is that a decline in PES leads to a corresponding decline in physical activity (PA), which contributes to an increase in hypokinetic diseases among school-aged children and adolescents. PES (a planned, sequential K–12 standards-based program with written curricula and appropriate instruction designed to develop the motor skills, knowledge, and behaviors of active living, physical fitness, sportsmanship, self-efficacy, and emotional intelligence), according to SHAPE America [1], has the potential to make distinctive contributions to the development of children’s fundamental movement skills and physical competences, as well as support the development of social skills and behaviors, self-esteem, and preschool attitudes, and in certain circumstances, academic and cognitive development, according to Bailey [2]. 

The overall goal of PES is to make its pedagogical approach of educating the body to be permanent by teaching children about movement and developing the necessary skills to become proficient in many kinds of PA, as stated by Guedes [3], as well as to develop the patterns and interest in PA, which are essential for healthy development and lay the foundations for adult healthy lifestyle, as reported by ICSSPE [4]. According to SHAPE America [1], the purpose of PES is to develop the motor skills, knowledge, and behaviors of active living, physical fitness, sportsmanship, self-efficacy, and emotional intelligence. In other words, UNESCO [5] elucidated that PES should be effectively implemented in order to provide a platform for broad social inclusion and develop the skills and knowledge necessary to define new forms of global citizenship. In this regard, UNESCO [6] proclaimed that the practice and full participation in PES is a fundamental human right for all. In this light, Wright et al. [7] substantiated that the school setting remains one of the conducive environments for promoting a physically active lifestyle among children and adolescents.

However, as stated by UNESCO [5], Weedon et al. [8], and Louis [9], PES is on the decline. PES declination has been strongly evidenced by UNESCO [10] in its survey conducted in all regions across the globe, revealing that (a) PES is being replaced by core subjects such as mathematics, the science subjects, language, arts, etc.; (b) PES-allocated curriculum time is being diverted to such core subjects; (c) PES teachers are being assigned other duties, such as logistics; and (d) PES is being replaced by cleaning or sending students home. On a related note, UNESCO [10] has stated that PES has lower esteem and status compared to other subjects. This was especially noticeable in North America, Africa, and the Middle East, with 77%, 69%, and 65%, respectively. Subsequently, the average time allocated to PES in primary and secondary schools remains low, i.e., 97 and 99 min, as against an ideal of 120 and 180 min in primary and secondary schools, respectively. Apart from insufficient curriculum time allocation, cancellation of PES lessons has also been reported to the extent of 100% in North America, 65% in both Africa and the Middle East, and 52% in Latin America/Caribbean, according to UNESCO [10]. 

On account of this PES downturn, the prevalence of global physical inactivity among children and youth has been observed to be particularly high. For instance, the findings of a research study conducted by Guthold et al. [11] reported that 81% of adolescents were not physically active, of which 77.6% and 84.7% were boys and girls, respectively. Another example of what Guthold et al. [11] meant is that observed by Kimm et al. [12], who reported a 100% and 64% decline in habitual leisure-time PA for African-American girls and White girls by the age of 16 or 17 years old, respectively. From this standpoint, it was noted that such a decline in PA increases with age, particularly in high-income countries, according to Hallal et al. [13] and Corder et al. [14]. More recently, Remmers et al. [15], Telama and Yang [16], and Caspersen et al. [17] published research studies that show that PA decline occurs between the ages of 12 and 13 years onwards. At this point, it is worth noting that Aubert et al. [18] observed that more than 70% of youngsters in various countries do not meet the PA level needed for a healthy life. Increasingly important is the fact that only 20% of the world’s adolescents are physically active, according to WHO [19]. 

In a similar vein, it has been pointed out that one in four, equivalent to 23% of adults, and three in four, equivalent to 81%, of adolescents aged 11–17 years do not meet the global WHO recommendations on PA for health, according to UN-Habitat [20]. 

In view of this emerging physical inactivity, sedentary health-based diseases and disorders, as well as the global health crisis, remain unresolved issues. According to Toschke et al. [21], chronic diseases have been particularly prevalent among children and adolescents, due to a lack of effective PA. To further clarify this, according to WHO [22] and Lin et al. [23], over 340 million children and adolescents aged 5–19 were classified as overweight or obese in 2016, while 476.0 million children and adolescents were diagnosed with diabetes mellitus in 2017. 

Increasingly, negative consequences in various domains such as physical (worsened bone density, strength, and flexibility), psychological (increase in the occurrence of major depression, poor concentration and self-esteem, negative bullying), and academic (decrease in standardized test grade) have also been reported by Rasberry et al. [24]. Above all, physical inactivity was ranked third among the six risk factors, accounting for 19% of global fatalities and 7% of global DALYs. Moreover, according to WHO [25], physical inactivity is responsible for 21–25%, 27%, and 30% of breast and colon cancer burden, diabetes, and ischemic heart disease burden.

In another example, WHO [26] reported that mental health conditions currently account for 16% of the global burden of diseases and injury in children aged 10–19 years old. In this light, depression has been identified as one of the leading causes of illness and disability among adolescents. Similarly, suicide is reported as the third leading cause of death in children aged 15–19 years old.

Physical inactivity is increasingly recognized as a serious, worldwide public health concern, especially among young people (school-aged children and adolescents). This is happening at a time when PES, which has been shown to be a single subject with the potential to provide the students with various benefits, including health-related ones, UNESCO [5] (p. 6) is on the decline. In fact, it is evident that the entire community is suffering from a physical inactivity epidemic, especially among young people. As such, research to date has tended to focus on PA rather than PES.

This current paper therefore seeks to remedy these problems by analyzing the significance of PES in various domains identified as cognitive, physical, affective, healthy, social, moral, cultural domains, and SDGs as well as raising an awareness of PES in order to encourage governments, organizations responsible for PES, and schools to translate promises into practice. 

## 2. Brief Background of PES

The starting point of PES can be traced back to the early societies, whereby their education philosophy was, according to Van Dalen and Bennett [27], education for survival. In this regard, the purpose of education was to ensure the survival of society. Thus, the curriculum was made up of courses such as hunting, throwing, running, jumping, etc., in line with strengthening the people to find food and protect their families against harmful animals and other disasters.

In a similar vein, the philosophical foundation of ancient Greeks on education was the notion of dualism, which, in the Greek curriculum, was featured under two components, namely gymnastics and academics, according to Laker [28]. In essence, education aimed to ensure the aesthetic and physical development of the body by means of sport; specifically, Sparta promoted PES by targeting military fitness, as opposed to the more holistic education for Athens.

During the Dark Ages, the aims of developing the body and mind equally that came from the Greek civilization, which considered the body as a partner or guardian of the mind and soul, became devalued. Later on, during the Renaissance (rebirth, discovery age), the development of a complete person as a priority was recovered by the Greeks, since such fully educated people were in need to take their place in a polite and cultured society. Hence, PES as a component of holistic education was in service of the needs of the society, according to Laker [28]. Until around 1820, much of focus of schools was on PES expressed in gymnastics, hygiene training, and care and development of the human body. By the year 1950, major courses in PES had been introduced in over 400 institutes to promote PES. 

Even though this was considered an outstanding progress, it did not lead to the success of PES as a legitimate subject in all schools worldwide. The evidence suggests that, later in the 20th century (1970s–1980s), PES suffered a strong decline that is associated with the increased availability of other subjects, whereby the attention, time, and values assigned to PES were shifted to academics, according to Excite Education [29]. 

Consequently, it was noted that both pedagogy professionals and practitioners failed to assume their responsibilities of clarifying the nature of the field at the school level and advocating for its restoration in order to address the PES crisis, as claimed by Guedes [3]. Realizing this crisis, UNESCO initiated and enforced the international charter of PES across the world on 21 November 1978.

With the PES decline, the International Council for Sport Science and Physical Education (ICSSPE) was established to tackle the problem at hand. It is in this context that the first international summit was organized on 3–5 November 1999 in Berlin by ICSSPE with support from the International Olympic Committee (IOC), United Nations Educational Scientific and Cultural Organisation (UNESCO), and WHO, bringing together policymakers, physical education practitioners from around the world, researchers, and administrators to share all necessary information concerning PES. 

Reporting his observations, Hardman [30] documented his findings from the international summit which reaffirmed the perilous position of PES to the extent that the UNESCO’s 1978 international charter of PES was found to be unimplemented. What is more, it was noted that PES was pushed into a defensive position under which it faced a reduction of curriculum time allocation, deficient resources (financial, material, and human), and marginalization associated with low value, status, and esteem by authorities. Until now, there has been a need to turn promises into realities and policies into actions if threats are to be vanquished and a convenient future for PES is to be maintained. 

## 3. Material and Methods

This review was conducted by adopting the external desk research method from Mangal and Shubhra [31] used to enhance the overall effectiveness of the research. For the purpose of this study, a comprehensive search was carried out to retrieve related reports, policies, charters, guidelines, international position statements, and support statements, as well as other relevant documents and articles. For the sake of documenting the analysis method, as well as inclusion criteria, a search protocol was designed in advance. In so doing, a search strategy for the identification of works in the relevant literature containing key terms in their title and abstract was developed. This search strategy was tailored to Google Scholar, ScienceDirect, PubMed, and Eric. The search terms used with Boolean Operators were “physical education and school sports” AND “cognitive OR physical OR affective OR healthy OR social OR moral OR culture” OR “Sustainable development goals”. 

The search focus was mainly on the existing English literature related to the role of PES in the field of social sciences and health sciences. Thus, it was narrowed to subject areas identified as PA, sports, recess, recreation, and dance. The researchers included study publications, reports, and data and information from census or other scientific data-collection procedures to ensure validity and dependability. Thus, in light of preventing personal bias, information and data collected from personal diaries, newspapers, and magazines were excluded from this study. Similarly, the researchers ensured that the relevant data were available before undertaking further stages of this study in order to avoid making assumptions about the availability of the required data.

## 4. Benefits of Physical Education and School Sports

The European parliament 2007 resolution [32] declared the following: “PES has the propensity to make significant and distinctive contributions to children, schools and wider society: respect for the body, integrated development of mind and body, understanding of PA in health promotion, psycho-social development (self-esteem and self-confidence), social and cognitive development and academic achievement, socialisation and social skills (tolerance and respect for others, co-operation and cohesion, leadership, team spirit, antidote to antisocial behavior) and aesthetic, spiritual, emotional and moral (fair play, character-building) development, a panacea for resolution of the obesity epidemic, inactivity crisis and sedentary lifestyle, enhancement of quality of life etc.”. 

PES, according to SHAPE America [1] creates a framework of life skills that shapes the whole person, encouraging smart choices and cultivating a healthy lifestyle, while both PA and effective PES are proven essential elements in the formative growth of children and adolescents, as well as an evidence-based approach to improving academics and benefiting students’ physical, cognitive, and mental health. The section hereunder therefore explored the role of PES under cognitive, physical, affective, healthy, social, moral, cultural domains and SDGs.

### 4.1. Cognitive, Academic Performance and Brain Health

A healthier body, academic performance, cognitive development, and lifelong brain health have all been linked to the time students spent participating in PA either as a one-time event or habitual. As a matter of fact, Plato, Aristotle, and Rousseau, the classical scholars of education in the 18th century, contended that the development of the body has to balance that of the mind [33].

To bring to light the issue of improved academic performance and cognitive development through PES, several studies have been carried out to establish the contribution of PES in improving students’ cognitive development, brain health, and academic achievements. 

As a matter of concern, improvement in measures of cognitive skills and attitudes are positively benefited from improved PA engagement level in PES. Hence, participating in a prolonged PA at school helps the students to increase their cognitive preparation processes because of a more effective working memory network, as reported by Boykin and Allen [34], Oja and Jürimäe [35], Reynolds and Nicolson [36], and Kamijo et al. [37].

In the same way, cognitive benefits such as executive function are accrued from PA participation no matter how long it lasts or how intense or frequent it is. Rather, Budde et al. [38] found that even a single occurrence of high-quality PA can improve children’s or teenagers’ executive function scores in an executive function test.

More importantly, Kramer et al. [39] bolstered that participating in PA improves not only cognitive development and academic performance of the students involved but also contributes significantly to maintaining healthier cognition in adulthood and even at old age. Thus, there is evidence that early childhood participation in PA helps in combating cognitive aging.

Equally important is the fact that improved academic performance has been closely associated with PA participation in conditions where students need to spend a certain period of time with a given intensity or in some cases frequently/repetitively.

In this case, Donnelly and Lambourne [40] established that regular participation in PES increases the students’ academic performance. This is evident in the case of Bartholomew and Jowers [41], who noted that better attention in the classroom, as well as on-task behaviors and concentration, is influenced by PES, which, in turn, results in improved academic performance.

The next similarity is an assertion made by Hillman et al. [42], emphasizing the function of PES in improving attention allocation and working memory to a single cognitive activity completed, regardless of the intensity and time constraints. A supportive view of Hillman et al.’s assertion was articulated by McNaughten and Gabbard [43], who stressed that even a short bout of PA equivalent to 30 min positively affects cognitive functioning in school-aged children.

Other researchers have also revealed that the positive effect of PES is more likely to be achieved provided that PA is delivered over a long period of time. In this regard, Gabbard and Barton [44] emphasized that a significant improvement in academic achievement such as mathematics performance is achieved through long participation in PA for at least 50 min. On a related note, the CDC [32] insisted that PES serves a positive impact on academic achievement if the overall PES time is increased.

In a similar light, it has been indicated that students’ executive functions such as attention and inhibition, healthy attentional process, perceptual skills, intellectual quotient, verbal tests, mathematics tests, memory, readiness, cognition, and emotional regulation and balance are increased when PES subject is given a high priority by allocating more time to engage students in moderate-to-vigorous PA, which results in overall academic performance, according to Sallis and Owen [45]; Verdine et al. [46]; Etnier and Sibley [47]; and Stevens [48].

As far as brain health is concerned in relation to PES, different researchers have conducted a variety of studies and come up with different views about the benefits of PA to brain health. PA affects the physiology of the students’ brain by increasing cerebral capillary growth, blood flow, oxygenation, production of neurotrophins, growth of nerve cells in the hippocampus, neurotransmitter levels, development of nerve connections, density of neural network, and brain tissue volume, according to Trudeau and Shephard [49], Hillman et al. [50]; and Rosenbaum et al. [51]. Greater attention, information processing, storage, and retrieval; improved coping and positive effect; and reduced cravings and pain sensations have all been linked to physiological changes in the brain.

Hills [33] argued that active engagement in PES improves academic performance by increasing blood flow to the brain, increasing mental alertness, enhancing mood, and increasing self-esteem. Consistent with the findings of Hills is the findings of Shephard [52], which stated that changes in cognitive functioning (increased blood flow into the brain, increased level of arousal, and stimulated brain development) are a reflection of any improvement in academic performance after engaging in PES.

Contrary to the above are the opposing views obtained from other studies undertaken to ascertain the cognitive, academic performance, and brain health benefits achieved through participation in PES; they revealed no relationship between these variables, even though the former determined the significant impact. Such contradictions are dependent on the dose prescribed to PES so as to offer the benefits ascribed to it.

Fisher et al. [53] argued that active participation in PES has no correlation with academic performance. Moreover, Ahamed et al. [54] found no significant difference between the treatment and control group in a standardized cognitive abilities test after 16 months of a classroom-based PA intervention under a cluster randomized trial.

In the same way, Tinning and Kirk [55] found no difference in academic subjects between the students who were allocated 90 min/day participating in PA and those who had not been engaged in such a program. Parallel to these opposing views, Melnick et al. [56] found no or a trivial correlation between active participation in PES and academic achievement.

Another point to note is the null findings that were revealed between the contribution of PA and the cognitive or healthier brain. According to the null findings, PES is established neither to harm nor to benefit the students with cognitive development, academic performance, and brain health while engaging in PES. In this specific instance, on the completion of his study, Bailey [57] noted that increased PES time does not negatively affect cognition. Moreover, Trudeau et al. [58] and Trudeau and Shephard [59] confirmed that PES has no ill effect on academic learning.

The aforementioned existing literature that we reviewed presented contradictory views about the contributing benefits of PA to cognitive development, academic performance, and brain health of the concerned students, whereby some researchers revealed a significant association between these variables, while others found no relationship, regardless of those that claimed null findings.

Due to this inconsistency, in contrast to several research studies that undoubtedly confirmed various benefits of PES, it is clear that robust longitudinal cause-and-effect research is needed to explore the role of participation in a particular PA on cognitive development, academic performance, and brain health, since disagreements remain rampant on whether the relationship between PA and academic achievement is causal. It is also clear that further understanding is needed to ascertain the level of intensity and duration that children need to reach so as to fully gain the cognitive benefits available by participating in PA. However, much work still needs to be performed in order to examine the appropriate type of physical exercises to be undertaken concerning culture, gender, and age level of students such as children and adolescents that can lead to cognitive benefits, since educational demands change as children and adolescents change. Therefore, PES should be one of the compulsory subjects that is allocated appropriate time on schools’ timetable to expose students to a planned exercises providing the students with the opportunity to gain such benefits regardless of dose or intensity.

### 4.2. Physical Domain

PA has been established as one of the leading factors influencing physical health by curbing the causes of diseases, reducing the risk of chronic diseases, enhancing efficient functioning of the body, and providing remedial benefits, as well as health-related fitness within childhood and adolescence; and it continues throughout adulthood and old age toward a satisfactory future life, according to Sallis and Owen [45]; Bailey [57]; and Fernandes and Sturm [60].

In essence, Bailey [57] emphasized that PES significantly benefits the participants with general health through efficient functioning of the body; the remedial benefits include the correction of poor posture and the developmental benefits such as assisting the natural pattern of growth of the child.

Consistent with the view of Bailey are the emerging points documented by several researchers who argued that participating in quality PES improves the physical status of the participants in terms of body mass index, resulting in a normal weight within the school period and in the future. Fernandes and Sturm [60] pointed out that effective participation in PES diminishes the potential for future mass increase among children. In their own words, Madsen et al. stated, “more physical education is associated with lower Body Mass Index scores” [61]. On a related note, Cawley et al. [62] made it clear that PES lowers both body mass index and the probability of obesity among grade-five male students. This was also exemplified in the work undertaken by Freedman et al. [63], who substantiated that engaging in quality PES from early childhood prevents obesity, which, indeed, starts at childhood and persists all through life, leading to the risk of being affected by hypokinetic diseases such as coronary heart diseases and diabetes.

Another supporter of PES and health-related fitness, Sdrolias [64], in his study undertaken in secondary schools, contended that quality PES results in a significant improvement in health-related fitness and psychological well-being in high-school students. Similarly, it has been noted that PES reduces the odds of being an overweight adult by 5% each day per week, while normal-weight children are 25% more likely to be normal-weight adults if they participate in PES at least five days per week, according to Mensschik et al. [65].

The most obvious and important benefit of active PA engagement is the significant improvement in health-related fitness components (aerobic fitness, muscle strength and endurance, flexibility, and body shape) in both school-aged children and adolescents, due to active PA participation. To bring this assertion to light, Chen et al. [66] examined the relationship between students’ physical fitness components and PA and noted that engaging in PES and recess, as well as sports/dance, significantly influences the overall health-related physical fitness. This finding is in line with the findings of the study conducted by Sallis et al. [67], who ascertained a significant association between the level of PA and health-related physical fitness among school-aged children and adolescents.

Unfortunately, PES, a single curriculum subject under which school-aged children and adolescents are supposed to gain opportunities to engage in quality Pas, UNESCO [5] (p. 6), has been mostly sidelined to the extent that physical inactivity has been declared one of the leading causes of death, disability, and insufficient quality of life, particularly in the Western world, according to USDHHS [68]. On the other hand, UNESCO [10] reported that PES is globally cancelled at 44%, despite the fact that it has been confirmed globally to be a compulsory subject, at 97%. This is a fact that indicates the inconsistency in translating policies into implementations. It is therefore clear that PES needs to be fully restored and maintained in schools by exposing the students to quality PES instruction within a recommended time depending on school level (elementary/secondary) or gender to serve its physical benefits to the students.

### 4.3. Affective Domain

Currently, affection is understood as a psychological and emotional well-being with associated components, namely mastery motivation, sense of autonomy, moral character, confidence, emotion, preference, choice, feeling, beliefs, attitudes, and appreciations, according to NRCIM [69].

At the same time, many affective benefits, such as happiness, enjoyment, and self-confidence, have been associated with active participation in PA. WHO [70], in its study about sports and children, validated that participation in PES improves self-esteem, self-perception, and psychological well-being of the participants.

As Gilman [71] has noted, the students who participate in PA experience more happiness compared to those who do not participate. A view that supported Gilman’s assertion is articulated by Bailey et al. [72], who pointed out that the 1909 syllabus clearly points out the affective outcomes of physical exercises as producing a cheerful and a joyful mood, as well as the expression of emotion. Some other interested researchers went further to determine the role of such happiness/enjoyment in future PA participation. Williams and Gill [73] and Sonstroem [74] reported that such happiness experienced within PA reinforces self-esteem, which, in turn, enhances further participation. Kimiecik and Harris [75] made it clear that such happiness also improves intrinsic motivation, which lowers anxiety, thus increasing participation.

Along the same lines, other studies have revealed some psychological benefits of PES participation. Mutrie and Parfitt [76] indicated that a positive correlation exists between PA participation and psychological benefits such as the reduction of stress, anxiety, and depression, as well as emotional growth and expression. Active engagement in PES reduces anxiety and depression and increases positive mood, self-esteem, and restful sleep, according to Dunn et al. [77] and Landers [78].

Although these aforementioned findings may be valid, a view that contradicts the former is that of Steptoe et al. [79], who rejected the opinion of a positive association between PA and affective domain of human development after he conducted a study across 21 countries which involved 16,000 undergraduate students. He established a negative correlation between PA, exercises, and depression symptoms.

After all, not much is known about the mechanisms by which such dimensions of affective development occur, according to Dishman [80]. Increasingly, Thirlaway and Benton [81] raised an existing confusion that it is unknown whether some forms of PA are more or less beneficial to the improvement of the affective domain than others. Whereas other arguments have rejected the idea that all groups experience psychological benefits from being active.

To this end, it is clear that PES needs to be resumed and should serve the students with all affective benefits discussed in the aforementioned literature. Although much research still needs to be performed in order to ascertain the genuine mechanism and appropriate form of PA that is more likely to serve affective benefits to the students, qualified, trained, and competent teachers are needed to instruct the students through some instructional curriculum models such as sports education, teaching personal and social responsibility, cooperative learning, etc., that are evidenced to promote the affective domain.

### 4.4. Healthy Domain

Earlier in the middle of the 20th century, PES targeting health-related fitness came into existence. This is undoubtedly due to the evidence that indicates the function of PES in improving the quality of life through its benefits to the muscles, bones, joints, heart, and mental health, just to mention a few, among school-aged children and adolescents who continue to adulthood and old age. In this regard, several studies have been conducted to find out the role of PES in maintaining health and preventing the causes of some diseases that emerge as a result of a sedentary health style.

According to the Institute of Medicine (IOM) [82], PA has several benefits in regard to various aspects of health, such as improved aerobic capacity, muscle and bone strength, flexibility, insulin sensitivity, and lipid profiles, resulting in the reduction of the risk of heart diseases, mental illness, and other chronic diseases, such as diabetes mellitus, osteoporosis, obesity, etc. These findings of IOM are in line with the findings of Bloomfield et al. [83], who carried out a research study on the role of PA on the life of the participants’ skeleton, bones, joints, and muscles. The findings of their study revealed that there is an increase in mineral accrual; an increase in bone strength which, in turn, reduces the risk of osteoporosis-related fracture; and, ultimately, an improvement in muscle strength, flexibility, coordination, and balance, as these are found to be significantly influenced by PA participation. A supportive view was observed in the study conducted by Masurier and Corbin [84], who reported that active participation in regular PA significantly reduced the risk of major chronic diseases such as heart diseases, high blood pressure, stroke, some forms of cancer, diabetes, and osteoporosis. On a related note, WHO [85] substantiated that PA enhances physical fitness in the areas of cardiorespiratory and muscular fitness; improves cardiometabolic health, particularly in blood pressure, dyslipidemia, glucose, and insulin resistance; improves bone health, mental health, and cognitive achievement; and reduces visceral adiposity.

More importantly, the literature shows that PA is beneficial to people of all ages, including children, adolescents, adults, and the elderly, provided people effectively participate in it. Hallal et al. [86] noted that the future morbidity (risk of fracture) is influenced by early PA, which is of great importance in the treatment, as well as the reduction in the rate and the severity of, some hypokinetic diseases in children and adolescents. Focusing particularly on children, the CDC [87] elucidated that engaging in PES and recess at school contributes much to improving cardiorespiratory and muscle fitness, as well as the promotion of a healthier body weight and body composition in children. Supporting this advancement of the CDC, the USDHHS [68], the CDC [87], and Bauman [88] asserted that a lower rate of chronic diseases such as coronary heart diseases, cardiovascular diseases, diabetes mellitus, hypertension, osteoporosis, and some types of cancer; and the reduction of premature death are some of the benefits adults gain due to actively engaging in PA.

Nonetheless, a controversy has erupted over the most effective PA dose, including the type, intensity, and frequency required to provide students with such health-related benefits. On the one hand, some scholars claimed that no matter how long, how intensely, or repetitively you engage in PA, benefits will be accrued. On the other hand, the researchers emphasized that there is need for a specific dose standard that must be met in PES so as to obtain the benefits accruable in PES. Of utmost importance is the fact that intense and frequent aerobic PA has been strongly evidenced to provide many health-related benefits.

Boreham et al. [89] and Imperatore et al. [90] ascertained that aerobic endurance corresponds with high-density lipoproteins, systolic and diastolic blood pressure, body mass index, measures of fatness and insulin sensitivity, and arterial stiffness. Associated with the views of Boreham et al. [89] and Imperatore et al. [90] are the findings of the experimental study undertaken by Davis et al. [91] which indicated a reduction in body fat among children and adolescents suffering from obesity or overweight when made to start aerobic exercises early in the program.

Taking into account the intensity and duration of aerobic PA, Baquet et al. [92] bolstered that regular moderate or vigorous intensified aerobic exercises undertaken within 30–45 min per session three days per week within three months resulted in increased cardiorespiratory endurance by 5–15% in youth. Similar to frequent PA, Corbin et al. [93] revealed that participating in PA improves immunological function and curbs the symptoms of arthritis, asthma, and fibromyalgia.

Masurier and Corbin [84] stressed that early PA in life acts similar to a vaccine for many diseases which attack the body later in life, and it also reduces the risk of diseases, thus improving the quality of life. Equally important are the health-related benefits from anaerobic physical activities, i.e., strength training or resistance exercises. In this case, Faigenbaum [94] established that anaerobic physical exercises positively enhance the quality of different aspects of the health of participants such as cardiovascular fitness, body composition, blood lipid profiles, and insulin sensitivity. Consistently, MacKelvie [95] insisted that strength training improves bone mineral density and bone geometry.

As far as PES and mental health are concerned, mental illness has been regarded as a global burden. This is because, by 2010, mental illness accounted for 15% of the global disease burden, according to Biddle and Mutrie [96] and Biddle and Asare [97]. Young people are particularly vulnerable to mental illnesses such as depression, anxiety, and the rest of the mental health disorders. Though mental illness may seem alarming, evidence has shown that PA can help to reduce and avoid mental illnesses such as anxiety and depression, as well as improve other elements of well-being, leading to long-term mental health, according to Ahn and Fedewa [98], and IOM [82]. Similarly, Ahn and Fedewa [98], Simms et al. [99], Biddle and Mutrie [96], and Dishman et al. [100] reported that active participation in PA lowers or reduces depression and its symptoms, anxiety and its sensitivity (a precursor to panic attacks and disorders), physiological distress, state of confusion, anger, and stress. It also improves mental health, dietary choices, and mood.

From the aforementioned literature we reviewed, PES has a substantial association with various aspects of health, including the body, skeleton, organs, and mental health.

In contrast, a sedentary health lifestyle is currently a major determinant of people’s health outcomes throughout their lives; an issue that could be linked to a lack of effective PES, which increases the risk of developing chronic diseases such as hypertension and coronary heart disease; mental health issues such as anxiety and depression; cancers such as colon and breast cancer; and even diabetes mellitus, osteoporosis, etc.

The crux of the matter is that, among the American adult population, 66% are overweight and 32% are obese. Approximately 19% of children and 17% of adolescents are overweight, and 37% of children and 34% of teenagers are either overweight or at risk of being overweight, according to Masurier and Corbin [84]. These facts indicate that several mandates that maintain PES as a compulsory and inclusive subject delivered to both boys and girls remain elusive. Therefore, there is need for a rationale to raise awareness about PES to be recognized as an important subject whereby trained PES teachers, materials and equipment, weekly time allocated to PES on the timetable, and an adequate budget are put in place to serve its purpose for school-aged children and adolescents.

### 4.5. Social Domain

PES is seen as a single bedrock subject that equips students with social interaction within this technological era, which is no longer providing the opportunity for people to meet and socialize, as it should naturally be. In some respects, students, to some extent, enjoy various opportunities of meeting and communicating, developing leadership skills, and ultimately learning social skills and behavior, while curbing, at the same time, the anti-social behaviors through PES.

In view of this perspective, Hellison et al. [101] indicated that participation in PES instils positive social behaviors in school-aged children and adolescents, such as cooperation, personal responsibility, and empathy. Afterwards, such participation in some circumstances helps in curbing current youth epidemics such as depression, crime, alcoholism, and drug abuse. In its recent report, SHAPE America [1] pointed out constructive competition, conflict resolution, decision-making, cooperation, and leadership assumption aspects as some of the benefits students gain through their interaction in PES.

In a similar vein, the Europe report asserted that only PES provides students with the opportunities of meeting and communicating with others and developing leadership qualities. More importantly, it instructs the participants about relevant social skills such as tolerance, respect for others, adjusting collectivism aspects including teamwork-spirit, cooperation, and cohesion, just to name a few, according to Svoboda [102]. Another emerging view which supports this assertion was articulated by Bailey et al. [72], who addressed the influence of PES on current global cleavage by arguing that PES has the potential to connect children of different social/economic classes and even those coming from different nations.

Of particular concern, the Qualifications and Curriculum Authority (QCA) [103] reported the constructive and corrective impact of PES, whereby it helps in improving students’ attendance, behavior, and attitudes within the school, as well as lowers the anti-social and criminal behaviors, according to Andrews and Andrews [104]. Indeed, the views of (QCA) and the Andrews corroborated with the assertion articulated by Sport England (SE) [105] that stated that participation of school-aged children and adolescents in PES assists them to gain social outcomes such as opportunities for active citizenship, increasing their attitude for learning as well as reducing youth crime and truancy.

In contrast to the social benefits ascertained by several researchers and scholars introduced herein, PES has been being devalued through different forms pushing it into a defensive position identified as (a) attributing low status to PES teachers; (b) assigning alternative duties to PES teachers such as logistics; (c) diverting PES time, which is already insufficient, to core subjects; and, in some schools, (d) replacing PES time with cleaning, etc., according to UNESCO [10]. As a consequence of this PES devaluation, students are still experiencing unpleasant social behaviors such as disrespect among themselves and some other related behaviors, such as truancy, absenteeism, alcohol and drug abuse, crime, and intolerance, just to name a few, as reported by Jean de Dieu and Andala [106].

Therefore, there is the need to call upon governments of nations to enforce PES in schools, as stated not only in international policies but also in their national PES policies such as to remedy the status of PES teachers through adequate continuous professional development (CPD) so as to update their pedagogical content knowledge (PCK) and current pedagogical models appropriate to serve social benefits such as teaching personal and social responsibility model, allow them to regularly teach PES following timetable, and make PES a compulsory subject with accountability for attendance and performance such as the other compulsory subjects so as to bridge the gap between agreements and actions.

### 4.6. Moral Domain

Moral behavior refers to activities conducted by following the rules which apply in a certain social context such as formal school/class rules, informal societal norms, and even the expectations related to behavior. Thus, moral values include honesty, fairness, fair play, justice, and responsibility, as reported by Wright and Taylor [107], Lumpkin and Stokowski [108], and Stoll and Beller [109]. According to this perspective, the existing evidence suggests that many moral benefits, such as experiencing moral socialization, moral values, ethical behavior, citizenship education, and social and moral characters, are accrued from participating in PES when students are given the opportunity to engage in an effectively planned PES.

A notable example of these moral benefits was found in studies undertaken by Bloom and Smith [110] and Sabock [111], who elucidated that PES provides the students with many opportunities to experience moral values such as cooperation, competition, role-playing, rules, regulations, and goal-based discipline. Moreover, PES assists in gaining self-discipline and order, manual dexterity, and even determination, according to Bloom and Smith [110], and Bailey [57].

In his own words, Sabock [111] (p. 271) argued that “the arena of sport can provide one of the greatest opportunities for a student to learn honesty, integrity, and ethical behaviour”. It is becoming increasingly important that PES has been proven to be a paramount subject, simultaneously instilling in the students social and moral characteristics such as cooperation with teammates; negotiation and creation of solutions against moral conflicts; development of self-control, fairness, and good work ethics; and displaying courage and learning of virtues such as teamwork, as reported by Shields and Bredemeier [112] and Weiss and Bredemeier [113]. The next likeness was the view articulated by Romance et al. [114], who argued that active participation in PES has been established as a source of positive moral socialization, and, to some extent, deliberate interventions in PES settings can improve moral conduct.

Another emerging feature of a moral aspect through PES is a view that effective PES has been indicated as a foundation for good citizenship. Engh [115] suggested that quality PES results in a good citizen education, which is, indeed, what PES teachers are supposed to teach in educational athletics as they teach other PES components. Supporting Engh, Raakman [116] substantiated that participation in PES could help develop engaged and balanced citizenship.

Despite the fact that PES positively influence the students’ moral development, the contrarians against this prevailing knowledge argued that PES participation may be a causal agent of negative moral development among participants, according to Bredemeier and Shields [117], Priest et al. [118], and Collin [119]. Another view that contradicts the view of a positive association between PES and moral education was found in the study conducted by Collin [119], who noted that unethical and aggressive behavior, which destroys the development and well-being of young athletes and the whole society, can be the result of a win-at-all-costs philosophy.

Despite these contradictions in moral benefits accrued from PES participation, it is important to note, however, that the quality PES delivered by professionally trained and qualified PES teachers adopting some of the current pedagogical models acknowledged to promote moral aspects of the students through their constructivism approach, including sports education, which focuses not only on playing roles but also duty roles, has been acknowledged to serve the needful under the moral domain. Thus, PES needs to be welcomed in schools to serve all moral benefits attributed to it.

### 4.7. Cultural Domain

UNESCO [120] defined culture as the set of distinctive spiritual, material, intellectual, and emotional features of society or a social group that encompasses not only art and literature but also lifestyle, ways of living together, value systems, traditions, and beliefs. In a similar vein, Zimmerman [121] made it clear that culture involves religion, food, language, marriage, music, dressing style, the dualism of what is right and wrong, rituals, ceremonies, etc.

In contrast to the other domains, finding existing works in the literature that addressed the contribution of PES to maintaining or improving the culture of a given society, turned out to be complex. However, some views have been pointed out by some relevant organizations and scholars, indicating that PES plays a significant role in encouraging school-aged children and adolescents to recognize and respect each other’s cultural characteristics, resulting in the prevention of some bad feelings such as extremism and racism, among others.

An example of this act was found in the International Charter of PES, UNESCO [6] which justified that the right and freedom of participating in PES should be granted without discrimination of any characteristics, including color, gender, language, religion, national or social origins, political or other opinions, property, birth, or other considerations. A supporting view of this assertion was put forward by Wright [122], who advised that PES teachers should not conceive that their task tool is technical; rather, they should aim at nurturing certain qualities required for a democratic society, such as self-confidence leavened by an agreeable humility, curiosity, courage, persistence, kindness, gentleness, care for the less fortunate, and care for other forms of life.

Before approaching the end of this cultural aspect, it is worth sounding a note of caution in the context that such a relationship can be bidirectional; that is, quality PES can help the students to learn and maintain their respective cultural characteristics and values while respecting those of others, resulting in a harmonious society. On the other hand, there is a possibility that some of such variety of cultural characteristics, e.g., religion, gender, dressing style, etc., may negatively affect PES participation at school.

In this regard, having completed their study about the influence of family and culture on PA among female adolescents from the Indian diaspora, Ramanathan and Crocker [123] revealed that female adolescents are not adequately participating in PA as males do. This was explained as due to the cultural belief that they are scared of losing their femininity while engaging in PA, and the issue of the belief that they need to stay at home supposed and be engaged with domestic duties. Similarly, religious belief is another example of a cultural characteristic that lowers the desire to participate in PA in certain societies. For example, female students from Muslim countries do not experience opportunities to effectively get involved in PA because of restrictions based on their culture, such as the dress codes; prohibited close contact with males; and lack of related facilities such as a prayer room, clean washroom with clean water, and women’s sport and fitness foundations [124].

The upshot of all of this is that some cultural characteristics and values are still preventing all school-aged children and adolescents from fully participating in PES, and this, in turn, violates the PES international charter of 21 November 1978, that allowed PES participation for all, without any kind of discrimination. Another emerging cultural aspect is the concern that some situations whereby PES is not given a top priority for its successful implementation can results in violation of cultural norms. To this end, all institutions responsible for PES should ensure adequate CPD for in-service teachers or supply trained PES teaches who have necessary PCK to help students with different cultures to learn regardless of culture differences.

### 4.8. PES on Sustainable Development Goals (SDGs)

It is important to signal our concern to the contribution of PES to the SDGs—a universal call to action that aims to create an equal and inclusive community with improved health by 2030. This ambitious plan consists of 17 goals with their corresponding 169 specific targets.

After the establishment of the SDGs, researchers in the field of education, particularly PES, conducted several studies to ascertain the contribution of PES in the context of SDGs and revealed that the majority of the SDGs can be achieved through the involvement of school-aged children and adolescents in quality PES. There is considerable evidence indicating that PES has a potential to create a favorable context which allows the promotion of different aspects associated with the development of the current SDGs, such as coeducation, entrepreneurship, cooperation, and respect.

The international conference of ministers and senior officials responsible for PES (MINEPS VI), UNESCO [125] established 9/17 and 36/169 goals and associated targets whereby sports-based approaches could make a significant contribution. To support the view of MINEPS VI, the study undertaken by Baena-Morales et al. [126] (pp. 7–10) explained the way in which 10/17 SDGs equivalent to 58.8% and 24/169 targets; that is, 40.5% could be achieved through PES.

Of little difference, Baena-Morales and Gonzalez-Villora, [127], who have made great strides in analyzing the role of PES to SDGs in three major dimensions, namely social, environmental, and economical dimensions, commented that SDGs should not be given much consideration as a reference, since they are too generic, but the specific targets make up SDGs.

Though some research studies raise a concern that the contribution of PES to the SDGs is slightly explored, according to Fröberg and Lundvall [128] and Baena-Morales and González-Víllora [127], others have explored the role of PA, sports, or exercises in general, Dai and Menhas [129]; focused their attention to the contribution of PES in relation to some selected SDGs, with particular aspects such as health and well-being partnership as explored by Lynch [130], it is clear that PES is a transcendental subject toward the achievement of SDGs, provided that it is given a top priority in schools worldwide. It is important to note that PES teachers should plan their lessons by linking the lesson instructional objectives with those of SDGs.

This paper provides an important opportunity to advance the understanding of the significance of PES in promoting a physically active health style in school-aged children and adolescents and the entire community, as well. It is therefore important to raise an alarm about PES enforcement to the governments of nations so as to empower PES in schools and make it serve its purpose for all students across the world.

## 5. Conclusions

PES has been evidenced to play a significant role in a holistic education to the extent of being considered as a backbone of the whole community in the 21st century, on account of the fact that school-aged children and adolescents are the ones that gradually become adults and later old people in their respective communities. That is to say, delivering quality PES to school-aged children is, at the same time, delivering an active lifestyle to the entire community throughout the life course. This is established based on the benefits obtainable from PES in all areas of human development, namely the cognitive, physical, affective, health, social, moral, and cultural aspects of human life, as discussed in this study.

The hindrances that impede PES from delivering all that it could offer to the school-aged children and adolescents which later affect the whole society include the following: (a) inadequately qualified teaching personnel; (b) insufficient time allocated to PES; (c) limited facilities, equipment, and materials; (d) deficit budget allocated to this subject; and (e) PE teachers detraction among others. Subsequently, a sedentary lifestyle has been mostly discussed as a pandemic among children and adolescents of this current century, resulting in suffering from hypokinetic diseases (coronary heart diseases, obesity, hypertension, osteoporosis, diabetes, etc.), as well as mental diseases such as depression and anxiety. Moreover, nowadays some students are still facing poor academic achievement, leading to increased repetition rate, drop-out rate, and ultimately on-time completion rate, an issue associated with the current sedentary lifestyle among students. From all such drawbacks of physical inactivity, one should wonder how perilous this coming society will be in the case that all of these challenges against quality PES remain unresolved.

To this end, it is important to raise these questions for the concerned leaders and related practitioners across the world, so as to come up with an effective and sustainable solutions. Apart from international charters, conventions, national policies, and international and national guidelines and endorsements, civil and private organizations (agencies) promulgated to address the promotion of PES. Considering also the fact that majority of parents’ perceptions support inclusive and quality PES for the benefits of their children, as well as the consequences of sedentary health style among all children, adults, and old people. Why are the governments of nations still inconsistent in their effort to convert their promises (agreements) of promoting PES into implementation/practice? Why are the governments of nations not willing to initiate mechanisms that aim to produce the required professionally trained personnel with the required PES resources and adequate budget? Why are school leaders still reducing or diverting allocated PES time to other subjects? Who would be held accountable for violating the universal right of quality PES for all and thwarting PES subjects from delivering all benefits claimed under its name?

“*Knowing is not enough, we must apply. Willing is not enough, we must do*”.

—Goethe [131]

## Data Availability

To prevent the assumptions of availability of required data, the authors needed to ensure the availability of required data before further stages of the study.
